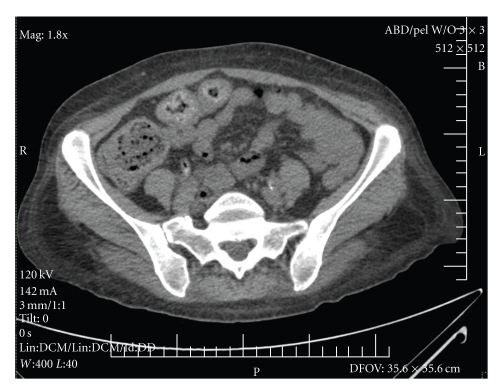# ACE Inhibitor-Induced Angioedema of the Bowel

**DOI:** 10.1155/2010/690695

**Published:** 2010-12-01

**Authors:** Tabitha Campbell, Bradley Peckler, Raleigh David Hackstadt, Austin Payor

**Affiliations:** ^1^College of Medicine, University of South Florida, Tampa, FL 33606, USA; ^2^Center of Advanced Clinical Learning, Emergency Medicine Residency, College of Medicine, University of South Florida, Tampa, FL 33606, USA; ^3^Department of Emergency Medicine, College of Medicine, University of South Florida, Tampa, FL 33606, USA; ^4^College of Osteopathic Medicine, Nova Southeastern University, Davie, FL 33314-7796, USA

## Abstract

Angiotensin converting enzyme inhibitor ACEI-induced angioedema of the intestine is a rare occurrence and often unrecognized complication of ACEI. We present a case of a 45-year-old Hispanic female with angioedema of the small bowel progressing to facial and oral pharyngeal angioedema. Patients are typically middle-aged females on ACEI therapy who present to the emergency department with abdominal pain, nausea, vomiting, and diarrhea. This is a diagnosis of exclusion, and physicians must have a high index of suspicion to make the diagnosis. Symptoms typically resolve within 24–48 hours after ACE inhibitor withdrawal. Recognizing these signs and symptoms, and discontinuing the medication, can save a patient from unnecessary, costly, and invasive procedures.

## 1. Introduction

Angioedema of the face and oral pharynx is a well-recognized complication of ACE inhibitor therapy. These medications can also cause angioedema of the bowel and may present a diagnostic dilemma to the emergency physician. Patients typically present with a complaint of abdominal pain with or without vomiting and diarrhea. Workup is usually nondiagnostic including leukocytosis and nonspecific bowel wall thickening on CT scan. Therapy consists of withdrawal of the medication. This is a diagnosis of exclusion, and physicians must have a high index of suspicion. Making the diagnosis can prevent patients from exposure to costly and invasive procedures. 

## 2. Case Report

A 45-year-old Hispanic female presented to the emergency department with a chief complaint of severe abdominal pain for the last several days that progressed to severe pain over the last 24 hours. Approximately one week ago, the patient was evaluated at another facility for a complaint of abdominal pain. Lab tests showed an elevated lipase, and the patient was diagnosed with pancreatitis. She was discharged home and instructed to follow a clear liquid diet and advance as tolerated.

The patient's past medical history was significant for hypertension, type 2 diabetes, chronic renal failure requiring dialysis, and the recent diagnosis of pancreatitis. Her medications included enalapril/hydrochlorothiazide, hydralazine, clonidine, metoprolol, metoclopramide, promethazine, mirtazapine, pantoprazole, insulin NPH/regular 70/30, alprazolam, and zolpidem.

On initial evaluation, the patient was complaining of several days of sharp, crampy abdominal pain worsening over the 24 hours prior to arrival. She complained of nausea, vomiting, and watery diarrhea for the past day. She denied fever, chills, dizziness, weakness, headache, chest pain, or shortness of breath. She denied blood in her emesis, stool, or urine. The remainder of the review of systems was negative. 

On initial physical exam, vital signs were significant for hypertension (175/86) and mild tachycardia (107). The patient appeared to be in acute distress secondary to pain. She was alert and oriented, oral pharynx was clear with no edema, erythema, or exudates, and neck was supple with full range of motion. Her lungs were clear to auscultation bilaterally. Cardiac exam was significant for tachycardia with a regular rhythm, no murmurs, rubs, or gallops. The patient's abdomen was diffusely tender and worse in the mid-epigastric and periumbilical region. She had no peritoneal signs, and rectal exam was hemoccult negative. The remainder of her physical exam was unremarkable. 

Initial labs were significant for an elevated white blood cell count of 19.8 K/UL with a left shift, and the rest of the hemogram was normal. Her metabolic panel revealed a BUN of 29 mg/dL and creatinine of 5.8 mg/dL (which was consistent with her baseline). Serum glucose was 182 mg/dL, amylase slightly elevated at 203 U/L (normal 36–128 U/L), lipase 43 U/L (normal 10–51 U/L), and liver function tests were normal. 

CT scan of the abdomen and pelvis was limited due to the lack of IV contrast with no significant findings other than mild edema of the small bowel, with No pancreatic swelling, fat stranding, fluid collection, free fluid in the abdomen, lymphadenopathy, or masses. 

The patient was given multiple doses of hydromorphone during her stay in the emergency department, with only minimal improvement of her pain. The patient was reevaluated on several occasions with no change in her physical exam. Shortly after returning from CT, the patient began complaining of difficulty swallowing and mild shortness of breath. Upon reevaluation at that time, the patient was found to have diffuse swelling of her face, neck, lips, oral pharynx, and tongue. The patient required emergent fiberoptic intubation and was admitted to the intensive care unit. During her hospitalization, C1 esterase inhibitor and complement levels were all within normal limits.

**Figure 1:**
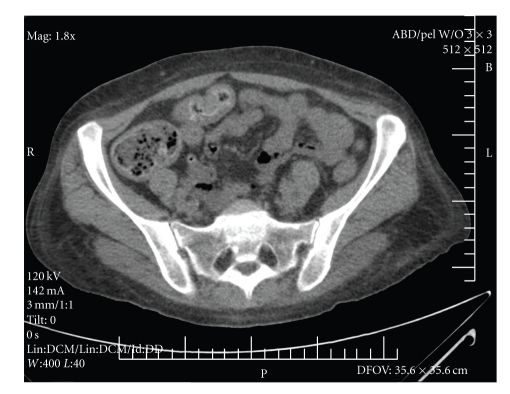


The patient was diagnosed with ACEI angioedema of the oral pharynx and small intestine. The ACEI was discontinued at the time of admission. The swelling improved, and she was extubated after 48 hours. The patient's abdominal pain resolved, and she was discharged home with instructions to avoid ACEI in the future. At followup visits over the next six months, the patient's abdominal pain had not returned (see Figures 1 and 2).

## 3. Discussion

After review of the English literature, we were able to find 21 documented cases of ACEI-induced angioedema of the bowel [1–10]. Patients typically present with unexplained abdominal pain despite extensive evaluation [1, 2]. The patient in this case initially presented with abdominal pain due to angioedema of the bowel. While in the emergency department, she progressed to angioedema of the face and oral pharynx, which is a rapid and atypical onset of angioedema from ACEI. 

Pancreatitis, obstruction, mesenteric ischemia, infection, cholecystitis among other abdominal emergencies, and C1 esterase inhibitor deficiency all need to be considered in the differential. Although the CT of the abdomen and pelvis was done without IV and oral contrast, which is recommended to fully appreciate pathology of the pancreas, it did not reveal any signs of pancreatitis. The patient's labs and CT scan were not consistent with a diagnosis of pancreatitis, obstruction, or acute infection. C1 esterase inhibitor deficiency was ruled out during her admission. As her facial angioedema resolved, so did her abdominal pain. The abdominal pain did not return after discontinuing the ACE inhibitor, leading to this diagnosis of exclusion. At 6-month followup, the patient remained free of abdominal pain. 

Approximately 30% of all ED visits for angioedema are from ACEI, while the annual rate of ED visits for ACEI-induced angioedema is 0.7 per 10,000 [11, 12]. Angioedema is asymmetrical nonpitting edema of the skin or mucus membrane and a well-documented side effect of ACEI [13]. ACEI-induced angioedema typically affects the face, eyelids, lips, tongue, neck, and pharynx, while urticaria or pruritis is seen only rarely [11, 13–17]. These adverse effects commonly present within the first 4 weeks after initiation of therapy and have not shown to be dose related or caused by one particular ACEI [13–16]. No definitive predisposing factors have been identified although the current literature suggests that patients with a history of either hereditary or idiopathic angioedema are at an increased risk for ACEI-induced angioedema [13, 15, 16]. Japanese patients have a lower incidence of angioedema from ACEI while several case reports have shown that patients of African origin have a significantly increased relative risk [13, 14, 18, 19]. 

The mechanism of action by which ACEI causes angioedema is not fully understood, but it is theorized to be from a biochemical rather than immunological reaction [16]. ACE converts angiotensin I to angiotensin II while also inactivating bradykinin. Increased levels of bradykinin, along with other mediators, are responsible for the angioedema reaction [13, 15, 16, 20]. Bradykinin causes vasodilatation and increased vascular permeability, thereby leading to angioedema. Some studies suggest that patients with a deficiency of aminopeptidase-P, another enzyme that catabolizes bradykinin, are at an increased risk of developing of angioedema from ACEI [13, 17, 20]. 

ACE inhibitor-induced angioedema of the intestines is a diagnosis that should be considered in any patient presenting with unexplained abdominal pain while on an ACE inhibitor. The incidence of ACE inhibitor-induced angioedema is low (0.1%–0.2%) with a small fraction of those representing angioedema of the bowel [1–4]. However, the exact incidence is unknown and is likely underdiagnosed [1, 4]. ACE inhibitor angioedema of the intestine is more common in females, with an average age of 48 years, suggesting a possible sex-linked or hormonal etiology [4, 9]. Common symptoms include abdominal pain, vomiting, diarrhea, and ascites [3–5]. Symptoms typically present within 24–48 hours of initiation of an ACE inhibitor, but there are case reports of facial angioedema 7 years after initiation of therapy [6] and bowel angioedema 9 months after initiation of therapy [3].

The management of ACEI angioedema should be prompt and aggressive with careful attention to airway management. Treatment can range from simple discontinuation of ACEI therapy to intubation and vasopressors, depending on the severity of the reaction. Angioedema of the intestine is reversible with cessation of the medication. Many of the patients discussed in the literature underwent invasive procedures including endoscopy, intestinal biopsy, exploratory laparotomy, and bowel resection, before a diagnosis of ACE inhibitor induced angioedema was made [1, 3, 4, 9]. Swift recognition is necessary to prevent unwarranted procedures, surgical intervention, or potentially death. Symptoms typically resolve within 24–48 hours after discontinuing the ACEI and continue to improve over the next 1 to 2 months [2, 10].

## 4. Conclusion

ACE inhibitor angioedema is a rare occurrence with intestinal involvement being less common. Unfortunately there is no specific test that can be used to diagnose the condition. Failure to make the correct diagnosis can place patients at increased risk of adverse outcomes due to invasive testing and procedures. While there does appear to be a greater risk of occurrence early in therapy, angioedema can present at any time and thus when treating any patient with abdominal pain use of ACEI therapy should be considered in the differential diagnosis. 

## Figures and Tables

**Figure 2:**